# Challenges for Service Robots—Requirements of Elderly Adults with Cognitive Impairments

**DOI:** 10.3389/fneur.2017.00228

**Published:** 2017-06-01

**Authors:** Agnieszka Korchut, Sebastian Szklener, Carla Abdelnour, Natalia Tantinya, Joan Hernández-Farigola, Joan Carles Ribes, Urszula Skrobas, Katarzyna Grabowska-Aleksandrowicz, Dorota Szczęśniak-Stańczyk, Konrad Rejdak

**Affiliations:** ^1^Department of Neurology, Medical University of Lublin, Lublin, Poland; ^2^Alzheimer Research Center and Memory Clinic of Fundacio ACE, Institut Catala de Neurociencies Aplicades, Barcelona, Catalonia, Spain; ^3^Department of Cardiology, Medical University of Lublin, Lublin, Poland; ^4^Medical Research Center Polish Academy of Sciences, Warsaw, Poland

**Keywords:** service robots, mild cognitive impairment, Alzheimer’s disease, user requirements, robotic assistant

## Abstract

**Objective:**

We focused on identifying the requirements and needs of people suffering from Alzheimer disease and early dementia stages with relation to robotic assistants.

**Methods:**

Based on focus groups performed in two centers (Poland and Spain), we created surveys for medical staff, patients, and caregivers, including: functional requirements; human–robot interaction, the design of the robotic assistant and user acceptance aspects. Using Likert scale and analysis made on the basis of the frequency of survey responses, we identified users’ needs as high, medium, and low priority.

**Results:**

We gathered 264 completed surveys (100 from medical staff, 81 from caregivers, and 83 from potential users). Most of the respondents, almost at the same level in each of the three groups, accept robotic assistants and their support in everyday life. High level priority functional requirements were related to reacting in emergency situations (calling for help, detecting/removing obstacles) and to reminding about medication intake, about boiling water, turning off the gas and lights (almost 60% of answers). With reference to human–robot interaction, high priority was given to voice operated system and the capability of robotic assistants to reply to simple questions.

**Conclusion:**

Our results help in achieving better understanding of the needs of patients with cognitive impairments during home tasks in everyday life. This way of conducting the research, with considerations for the interests of three stakeholder groups in two autonomic centers with proven experience regarding the needs of our patient groups, highlights the importance of obtained results.

## Introduction

According to the World Health Organization (1), dementia is one of the major causes of disability and dependency among older people.^1^ Altering the way an older person moves around the house, manipulates objects, and obtains sensory perception of the surrounding home environment exerts negative effects on their capacity to execute daily home activities on their own. Such effects are magnified in the case of persons with cognitive impairment.

A person with amnestic mild cognitive impairment (MCI) and early stages of Alzheimer disease (AD) has difficulties in instrumental activities of daily living, which depend on memory and executive functioning (2–4).

With the progress of the disease, the help needed for the execution of daily tasks normally increases, leading to a burden on the shoulders of informal caregivers, and in many cases to institutionalization (5).

The number of elderly adults and the incidence of cognitive impairment among them are increasing with the proportion of people aged over 60 being expected to double between 2,000 and 2,050 (6).

As a result, the resources allocated to assisting elderly people will not prove sufficient in the foreseeable future. Robotic assistants could be a way to help people remain healthy and safe in their own homes, ensuring their independence in everyday life. In this context, several social robots, which are human or pet-like robots such as NAO, Paro, KASPAR, PaPeRo, AIBO, and iCat aim at providing social support, engagement, and independence for people with special needs (7–11). Thus, people with cognitive impairment constitute a group, which may particularly benefit from healthcare robots. Currently, the achievements of social robots to date is being projected to achieve psychological and physiological improvement of cognitive impairment conditions among older people and others (8, 12). It also enables older people to become more independent (13–15). The 5-year-long observational study of interaction between robots and 139 people with dementia suggests that robots can improve the quality of care for people suffering from dementia (16). Additionally, the demand for social robots is expected to improve the capacity of caregivers in performing daily activities (17–19). A social robot can significantly improve the quality of social services by assisting caregivers (20–24). Some studies identify key areas of needs to be met for persons with dementia (25–27), but studies investigating the needs in relation to robot usage in supporting older adults with cognitive impairment are sparse.

The main objective of this study was to identify user needs and try to classify these findings, which may be a forward step toward changes in robotics for senior adult assistance in cases of amnestic MCI and at early stages of progressive dementia. Carefully assessing needs and matching these to the provided technology can result in higher acceptance rates (28). The study was carried out within the framework of RAMCIP project (full name: robotic assistance for MCI patients) founded by European Programme Horizon 2020. The aim of the project is to create a robot that might support older adults with MCI living independently at home.

## Materials and Methods

This study was carried out in accordance with the recommendations of the Ethics Committee of Medical University of Lublin and the Fundació ACE Research Ethics Committee and also under supervision of Ethics Advisory Board established for RAMCIP project needs. Each participant of the focus groups signed written informed consent form in accordance with the Declaration of Helsinki.

Participation in the survey was completely voluntary and informed consent was implied. The survey questionnaires were fulfilled anonymously. All data and results from the questionnaires were presented maintaining participants’ individual privacy.

### Focus Groups

At the beginning, we held focus groups in two centers in Poland and Spain.

The workshop with medical staff, conducted in Poland in March 2015, was attended by seven medical doctors and one nurse from a Neurology Department with experience among patients suffering from cognitive impairment.

At the same time, a workshop for caregivers was conducted in Spain at the Diagnostic Unit of the Fundació ACE, Barcelona Alzheimer Treatment and Research Center; with 10 participants.

Focus groups were conducted by a moderator who followed the prepared plan. Participants exchanged their ideas based on their experience and knowledge.

### Surveys

The data collected during focus groups were used to create the survey questionnaire for medical staff, potential users, and their caregivers.

The answers from surveys were sourced from two autonomic centers: Medical University of Lublin (LUM) and Fundació ACE (ACE). The group of medical staff included doctors, nurses, psychologists, and therapists who were selected from the two centers based on qualification criteria, such as the experience with patients suffering from cognitive impairments. The potential user group included persons aged between 55 and 90 years with MCI or early stages of AD defined by MMSE score of 20–26 points. The third group in that selection was related to potential user groups, as these participants were responsible for taking care of the patients. In the case where many caregivers took care of a patient, the respondent group included the most involved and closest ones to the patients (e.g., family members).

Participants were randomly selected for the study basing only on the time of their arrival at the centers (LUM and ACE) during April–June 2015. In total, we received 264 completed questionnaires (100 from medical staff, 81 from caregivers, and 83 from potential users). One hundred fifty-four surveys (50 from medical staff, 51 from caregivers, and 53 from potential users) were gathered by ACE; the remaining 110 surveys were collected in Lublin (50 from medical staff, 30 from caregivers, and 30 from potential users). Table 1 presents the percentage values for gender distribution in the tested group. At least 2/3 of respondents were women.

**Table 1:** **Gender distribution (in %) in the tested group**.

	GENDER			
	LUM		ACE	
	Male	Female	Male	Female
Potential users	10 (33.33%)	20 (66.67%)	21 (39.62%)	32 (60.38%)
Caregivers	6 (20%)	24 (80%)	20 (40%)	30 (60%)
Medical staff	12 (24%)	38 (76%)	8 (16%)	42 (84%)

A brief characteristic of the group of cognitive impairment patients is shown in Table 2. Among potential users 16 (19.28%) were AD patients and 67 (80.72%) were MCI patients.

**Table 2:** **Brief characteristic of the group of cognitive impairment patients**.

	Alzheimer disease		Mild cognitive impairment	
	LUM	ACE	LUM	ACE
Male	3	7	8	13
Female	4	12	15	21
Education level	Elementary (2)	Elementary (6)	Elementary (7)	Elementary (11)
	Secondary (4)	Secondary (10)	Secondary (12)	Secondary (17)
	Higher (1)	Higher (3)	Higher (4)	Higher (6)
Positive attitude toward new technologies	81.01%			

The issues included in the surveys, as well as their general layout, were similar for all stakeholders. The survey questionnaires included questions related to four sections: functional requirements; human–robot interaction, the design of the robotic assistant and social acceptance aspects.

The questionnaire layout was fully prepared by the authors of the survey and included close-ended and open-ended questions in each section. The data analysis shown in the tables are the results of the interpretation of the robot role from the stakeholders point of view. The data analysis shown in the Tables 1–6 was performed based on the answers given to closed-ended questions.

Based on the answers from close-ended questions, we specified prioritization of the stakeholders requirements and needs regarding robotic assistants. The requirement with high level priority means that a robotic assistant must have this function, medium level—should have, if possible, and low—not necessary for implementation.

- High priority—answers that were chosen by over 50% of the respondents and pointing to a more than average necessity of functional requirement.
- Medium priority answers marked by 25–50% of the respondents and pointing to average necessity of functional requirement.
- Low priority—answers chosen by less than 25% of the respondents and pointing to a less than average necessity of functional requirement.

The layout of the questionnaire has caused the necessity to use two methods of prioritization.

The first method of prioritization was performed using the Likert format of answers in surveys. It was done with data regarding the prioritization of different capabilities of robotic assistants with regard to human interaction, where stakeholders had a possibility to choose one out of five answers. It implies that the scores are valued as follows: 1 (very important, a patient has substantial difficulties with this and the proposed solution is desirable), up to 5 (very unimportant, a patient can do this on their own without any difficulty and the solution is not desirable) (29). Next transformation based on the % from 1 (80%+ difficulty) to 5 (0% difficulty) was done After that the prioritization was conducted as follows: high priority mean less than 2, through medium priority between 3 and 2, up to low priority more/equal than 3.

In the part devoted to identification of main situations when robotic assistants could be helpful, where stakeholders had opportunity to choose one of three answers, the answers were categorized into three groups: must, might, should not, and then prioritization was performed regarding to the frequency of answers according to general prioritization assumption described above.

Regarding prioritization of the way in which robotic assistants should be operated, simple analysis was performed based only on the frequency of answers according to general assumption.

In order to present accurately the differences in requirements for the robot between potential users, caregivers, and medical personnel, answers from both open-ended and close-ended questions were used (Table 7).

Visualization in diagrams was also used for the effective presentation of the outcomes.

## Results

### Focus Groups

One of the main goals of the focus groups was to define a list of daily tasks for which a certain level of quality must be maintained or even improved by the robotic system and which are crucial for supporting life quality of potential users. Furthermore, general safety rules and social acceptance conditions of human–robotic assistant interaction were explored.

As the main problems and challenges in MCI and AD patient care the medical personnel highlighted problems with: regular medication intake, task accomplishment, and general forgetfulness.

In comparison to medical personnel, the caregivers focused on issues connected with daily care and hygienic procedures: dressing and changing clothes, proper nutrition, bathroom usage, and other daily activities like cooking, shopping, and laundry.

### Surveys

#### Social Acceptance Aspects

Social acceptance of robotic assistants reached a high level among all groups of respondents. Most of them believe that a robot may be really helpful during daily life routines. Over 80% of the respondents think that it is good idea to replace a human caregiver with a robotic assistant. Only fewer than 20% of respondents think that it is questionable, because robots never fully replace humans. More than 75% of caregivers agreed to leave the potential user alone with a robot. However, caregivers (83, 95%) similarly as potential users (69, 88%) need minimum 3–4 training sessions before they agree to cooperate with a robotic assistant.

Over 60% of potential users would like to treat robotic assistants as friends, which means that they would like to personalize it and show the robotic assistant to their family and friends. It is necessary for users to feel safe with a robotic assistant. That is why 73.5% of respondents wish the robotic assistant was able to call for help if something bad happens to them and even 53% of them would be ready to move some furniture to let a robotic assistant move everywhere. It is also important for them whether the robotic assistant has a stand-by function even during the night.

#### Functional Requirements

As shown in Table 3, in order to define the overall priority level of each requirement, the priority levels that were attributed to them by each group involved in the surveys were taken into account. Functional requirements are categorized as high, medium, and low priority. The first seven functionalities listed in the table have high priority for the respondents. These functionalities are especially associated with the potential user safety, like calling for help if something happens to the patient or with general memory support by reminding the users about medication intake or about boiling water, turning off the gas and light.

**Table 3:** **Prioritization of functional requirements**.

The level of support offered by a robotic assistant during activities of everyday life	Frequency of responds (%)	Users priority	Frequency of responds (%)	Caregivers priority	Frequency of responds (%)	Medical staff priority	Frequency of responds (%)	Overall priority
Calls for help, if something happens to the patient	64.27	H	79.01	H	87.02	H	77.41	H
Detection of obstacles on the floor to prevent falls	57.83	H	58.02	H	68.64	H	61.98	H
Reminds the patient about boiling water, turning off the gas and lights	55.42	H	69.14	H	82.15	H	69.75	H
Monitors correctness of the patient’s medication intake	51.81	H	64.20	H	93.17	H	71.28	H
Reminds the patient that it is time for him/her to take his/her medication	50.60	H	74.07	H	89.12	H	72.39	H
Provides cognitive exercise to the patient	38.55	M	54.32	H	88.93	H	62.47	H
Finds things the patient is looking for	51.81	H	43.21	M	45.16	M	46.65	M
Is able to reach medication, which is difficult to reach for the patient	46.99	M	54.32	H	48.43	M	49.78	M
Reminds the patient that it is time for his/her meal or time to drink something	24.50	L	54.32	H	67.15	H	49.80	M
Stimulates the patient to keep in touch with family and friends	24.10	L	53.09	H	62.19	H	47.42	M
Reaches for fallen utensils and hands them over to the patient, in order to prevent the patient from bending over. Grasps things from the floor/shelves	45.78	M	38.27	M	44.13	M	42.85	M
Provides physical exercises to the patient	39.76	M	43.21	M	63.22	H	49.70	M
Reminds about important dates such a birthdays and medical appointments	42.17	M	41.98	M	61.29	H	49.35	M
Robotic assistant recognizes strangers and informs family members about such visits	40.02	M	61.14	H	81.88	H	62.36	H
Recognizes when it can or cannot open the house door	39.76	M	60.49	H	80.01	H	61.37	H
Helps the patient prepare food	24.30	L	23.46	L	65.17	H	39.52	M
Helps the patient put on clothes	24.91	L	32.10	M	43.26	M	34.07	M
Helps the patient take on/off her/his shoes	22.89	L	29.63	M	51.36	H	35.74	M
Helps the patient with a shopping list	21.69	L	24.57	L	17.52	L	20.99	L
Reminds the patient about TV programs	16.87	L	24.69	L	22.13	L	21.26	L
Helps the patient to clean the house	25.37	M	24.63	L	24.79	L	24.92	L
Helps the patient to put her/his feet on the footrest	12.05	L	30.86	M	11.87	L	17.75	L

Functions with medium priority regarding basic daily activities include preparing food and dressing. Also, robotic assistants might be helpful in reaching, grasping, finding things, and reminding of task ordering, which helps cognitive impairment patients compensate for their shortcomings.

Low priority functionalities regarding entertainment, relaxation and shopping.

#### Proactively (Autonomously) vs. on Demand

Memory alteration significantly decreases the quality of life in many aspects of daily living. In order to reduce the disease progress of MCI and AD patients, medical personnel emphasized that most of everyday life activities should be done independently. That is why robotic assistants should motivate and encourage the primary user to stay active. However, we cannot forget about the safety of the users, which is why in specific potentially hazardous situations robotic assistant must be able to take action autonomously, e.g., prevent falls (detect obstacles) or in the case of operating electrical and gas devices without the user’s attention. General findings show that the functions implemented into the robotic assistant and the ones that are correlated with the patient’s safety must be performed autonomously. At times, a potential user might not be able to do something alone (loss of consciousness) or may not realize or just forget that something important (e.g., medications intake) must be done.

Both groups (medical staff and caregivers) agreed that in unexpected and serious events such as falls, loss of consciousness, a robotic assistant should autonomously alarm relevant services and relatives.

Activities that should be done exclusively on demand are: carrying heavy things, preparing food, reminding about important dates and medical appointments, dressing, turning off a TV/radio, and reading a book. These functionalities or activities do not have direct influence on the potential user’s health and safety.

#### Human–Robot Interaction

Table 4 shows prioritization of the types of human–robot interaction. As a result of the analysis regarding the mode of human–robot assistant interaction, a “high priority” level was assigned to voice-operated system. It seems reasonable to expect it because it is a human natural and simplest way of communication. Medium priority was assigned to simple gestures, remote control, and touch screen. These ways of communication are widespread in new technology of electronic devices like TV sets, smartphones, tablets, etc.

**Table 4:** **Prioritization of the way in which robotic assistants should be operated**.

The way robotic assistants should be operated	Percentage of positive responses (%)	Users priority	Percentage of positive responses (%)	Caregivers priority	Percentage of positive responses (%)	Medical staff priority	Overall priority
By simple voice commands (voice operated system)	61.45	H	85.19	H	89.57	H	H
By touch screen	30.12	M	32.10	M	47.14	M	M
By simple gestures	27.71	M	28.40	M	64.31	H	M
By a remote control	25.30	M	27.16	M	31.16	M	M
By keyboard/buttons	3.61	L	7.41	L	9.19	L	L
N/A	12.05		3.70		5.17		L

The way of interaction is one of the most important factors influencing the degree of robot acceptance. Table 5 presents prioritization of different capabilities of a robotic assistant in relation to human interaction. It is important to all respondents that potential user may interact with robotic assistant, it means that:

**Table 5:** **Prioritization of different capabilities of robotic assistants with regard to human interaction**.

How much would you like the following capabilities to be implemented by a robotic assistant	Mean	Frequency of responds (%)	Users priority	Mean	Frequency of responds (%)	Caregivers priority	Mean	Frequency of responds (%)	Medical staff priority	Overall priority
The robotic assistant can reply to simple questions (e.g., what time is it?)	1.97	60.65	H	1.71	65.79	H	1.84	63.22	H	H
The robotic assistant can listen and respond to simple commands you give	2.02	49.7	M	1.50	70.00	H	1.60	64.85	H	H
The robotic assistant can comprehend and respond to simple gestures you make	2.08	48.44	M	1.72	65.88	H	1.69	62.16	H	H
The robotic assistant can take part in dialog interactions with the user in order to complete required tasks	2.09	48.13	M	1.79	64.21	H	1.77	61.17	H	H
The robotic assistant can talk to you regarding its current task/state	2.17	46.51	M	1.69	66.22	H	1.67	61.37	H	H
The robotic assistant can be easily controlled by the touch screen which is mounted on it	2.16	46.88	M	2.32	42.96	M	2.25	46.92	M	M
The controls shown on the touch screen of the robotic assistant change to reflect the needs of the user and the current task	2.2	46	M	2.14	49.26	M	2.15	49.63	M	M
The robotic assistant can display information on a touch screen that is mounted on it	2.23	45.48	M	2.14	49.3	M	2.17	49.39	M	M
The robotic assistant can project images and information on surrounding environment objects. floors, and walls or even body parts	2.39	42.26	M	2.38	44.43	M	2.02	45.35	M	M
The robotic assistant can be controlled directly through the touch screen it carries without the need to engage in a dialog with the user	2.64	37.24	M	2.45	45.7	M	2.25	43.47	M	M
The robotic assistant has a face that can express its feelings throughout the interaction with the users	2.76	34.76	M	2.59	40.22	M	2.44	39.49	M	M
The robotic assistant should continuously listen to the user for commands	2.03	49.38	M	1.42	71.58	H	1.54	65.48	H	H
The robotic assistant can understand the psychological state of the user and provide positive affective impact (actions)	2.39	42.2	M	1.90	61.92	H	1.83	57.06	H	H

- the robotic assistant can reply to simple questions,
- the robotic assistant can listen and respond to simple commands given by the user,
- the robotic assistant can comprehend and respond to simple gestures made by the user,
- the robotic assistant can take part in dialog interactions with the user in order to complete required tasks,
- the robotic assistant should continuously listen to the user for commands,
- the robotic assistant can talk back to the user regarding its current task/state,
- the robotic assistant can understand the psychological state of the user and provide positive affective impact (actions).

#### Design of the Robotic Assistant

The majority of the respondents would like a robotic assistant to have an anthropomorphic appearance (woman or neutral) and a face with positive emotional expressions. The material from which the robotic assistant will be made does not matter for the respondents.

**Figure 1:**
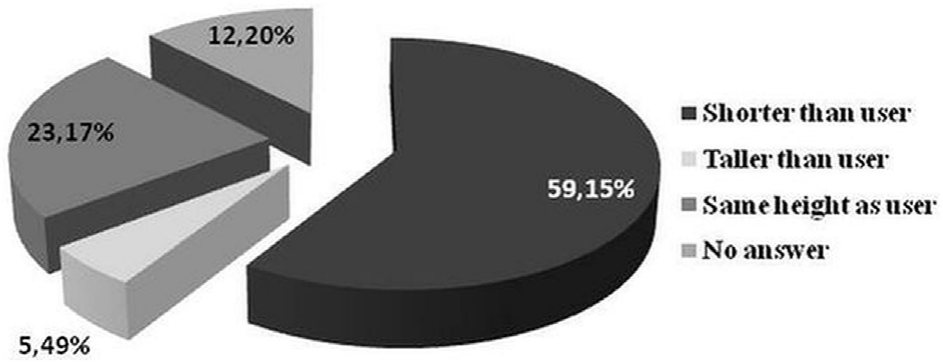
**Percentage distribution of respondents’ preferences regarding the height of the robot**.

**Figure 2:**
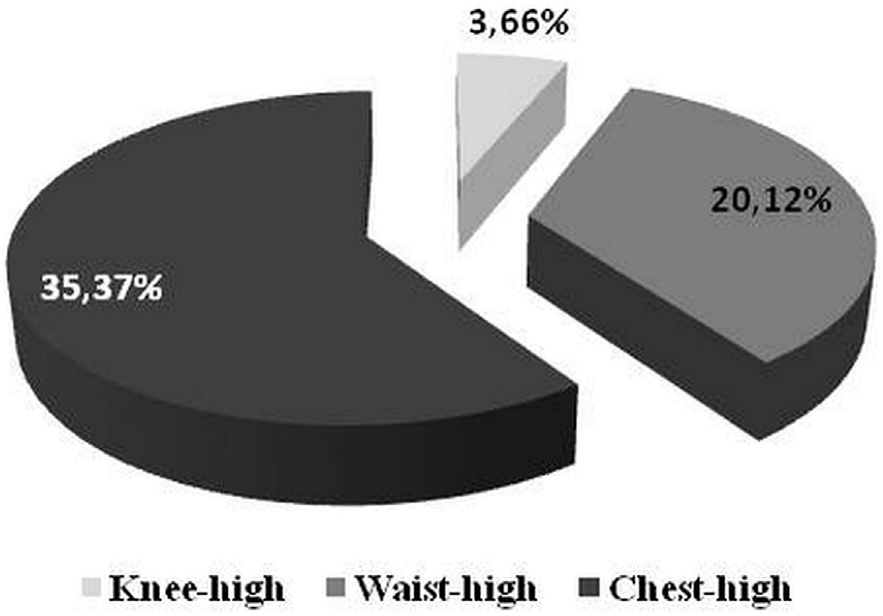
**Percentage distribution of prevailing answers from Figure 1**.

Most of the respondents think that the robotic assistant should be shorter than the user and the best height is approximately chest height. Table 6 and Figures 1 and 2 show the percentage distribution of respondents’ preferences regarding to height of the robot.

**Table 6:** **Percentage distribution of respondents’ preferences regarding to height of the robot**.

Height of roboticassistant		Potential users (%)	Caregivers (%)	Both groups (%)
Shorter than me: 59.15%	Knee-high	3.61	3.70	3.66
	Waist-high	19.28	20.99	20.12
	Chest-high	34.94	35.80	35.37
Taller than me: 5.49%	Up to 20 cm	6.02	4.94	5.49
	More than 20 cm	–	–	–
	The same height as me	19.28	27.16	23.17
	N/A	16.87	3.70	12.19

#### Differences in Prioritization among All Groups of Respondents

As it is shown in Table 7, there are some requirements important for all groups of respondents. They are associated with the safety of users during daily routine activities. Potential users belong to the group with the fewest number of requirements, which must be implemented into a robot (high priority functionalities). They indicated only six high priority functionalities opposite to 10 pointed by caregivers and 20 important functionalities listed by medical staff. The paternalistic approach of caregivers and medical personnel regarding the functionalities that must be implemented in the robotic assistant is the result of fears raised in open-ended questions as to whether the robot will be able to fully support human caregiver. They need to be sure that there are no doubts about leaving the patient alone with the robot. What is more, the medical staff would like the robot to possess functionalities, which might slow down or even induce a decrease in cognitive impairment of potential users.

**Table 7:** **The differences in approach to robot functionalities in which robot must be equipped between caregivers, potential users, and medical staff**.

Medical staff	Caregivers	Users
Informs about the danger arising from improper object location, Reaches for fallen utensils and hands them over to the patient, in order to prevent the patient from bending over, Register of falls, Reminds about returning to household tasks after being interrupted, Suggests which tasks are prioritized, Fetches things patient asks for, Stimulates the patient to keep in touch with family and friends, Reminds about the time for preparing meals, Suggests appropriate diet, Helps the patient prepare food, Informs family members about visits, Explains how to perform cognitive and physical exercises, Increase/decrease the difficulty of cognitive exercises. Reminds about technical aids that improve user mobility Provides instructions on how to use medical equipment Monitors proper selection of clothes, Points out improper selection of clothes, Helps in dressing up, Remembers where important items were placed, Reminds about events/deadlines.	Calls for help, if something happens to the patient, Reminds the patient that it is time for him/her to take his/her medication, Monitors the correctness of the patient’s medication intake, Detects obstacles on the floor to prevent falls, Reminds the patient about boiling water, turning off the gas and lights, Is able to reach medication, which is difficult to reach for the patient, Stimulates the patient to keep in touch with family and friends, Reminds the patient that it is time for his/her meal or time to drink something, Recognizes when it can or cannot open the house door, Provides cognitive exercise to the patient.	Calls for help, if something happens to the patient, Reminds the patient that it is time for him/her to take his/her medication, Monitors correctness of the patient’s medication intake, Detects obstacles on the floor to prevent falls Reminds the patient about boiling water turning off the gas and lights, Finds things the patient is looking for.

#### Differences between Polish and Spanish Respondents

There are some differences in prioritization among Polish and Spanish respondents regarding functionalities. The most noticeable differences are shown in Table 8.

**Table 8:** **The differences in prioritization among Polish and Spanish respondents regarding functionalities of robotic assistant**.

Functionalities	Prioritization of functionalities			
	%	Poland	%	Spain
Stimulating the patient to keep in touch with family and friends	20.64	Low	38.10	Medium
Finding things the patient is looking for	24.71	Low	53.95	High
Reminding about important dates such a birthday and medical appointments	19.68	Low	51.69	High
Reaching for fallen utensils and handing them over to the patient in order to prevent the patient from bending over. Grasping things from the floor/shelves	23.25	Low	50.92	High
Helping the patient clean the house	17.00	Low	38.18	Medium
Helping the patient properly button her/his clothes	17.89	Low	31.86	Medium
Helping the patient take off her/his shoes	23.01	Low	32.72	Medium
Helping the patient put her/his feet on a footrest	16.85	Low	26.54	Medium
Fetching things, the patient asks for	19.68	Low	39.69	Medium
Helping the patient draw up a shopping list	12.95	Low	33.47	Medium
Providing physical exercises for the patient	32.18	Medium	50.40	High

All the functionalities listed in the table above and implemented into the robot have higher priority for the Spanish respondents than the Polish ones. Survey analyses show that the Polish respondents have lower expectations regarding the robotic assistant than their counterparts from Spain.

## Discussion

This study, using a multimodal approach (focus groups, survey questionnaire) among three groups: older people with AD and early dementia stages, caregivers and medical staff, determined the desired requirements of the cognitively impaired patient, which may be met by a robotic assistant and would be enough to introduce robots as home care assistants. In this sections, the findings are discussed with respect to their levels of priority and with reference to the literature.

The most important capability of assistive technology is to handle emergencies in a private home. Robotic assistants have to recognize life-threatening situations by ongoing monitoring of health parameters, detecting falls and detecting dangerous situations in the household environment (e.g., working electrical or gas appliances). It is also important that robotic assistant must be able to inform appropriate support units and relatives in the case of emergency situation. Emergency alarms are a key point of robot safety, especially for social robots, since these robots aim to prevent any critical event for older people (8, 14, 30).

In this respect, the management of critical situations is one of the most preferred functionalities. The most common sources of home accidents leading to morbidity and mortality are falls and cardiovascular diseases (31–33). Along the same line of reasoning, our participants identified that a high level of priority, as a safety consideration, should be assigned to the removal of clutter and obstacles that may pose trip hazard.

Moreover, it is very important that robots are able to detect dangerous home environment situations regarding working electrical or gas appliances left uncontrolled. People with dementia who are living at home demonstrated that those living alone were perceived to be more at risk than those living with someone, and the most commonly reported risk is the one of fire (34).

People with dementia are at risk from self-neglect, which means that vulnerable adult is unable to exercise basic self-care (25). Signs of self-neglect include dehydration, malnutrition, untreated medical conditions, poor personal hygiene, unsafe or unsanitary living conditions, inappropriate or inadequate clothing, and inadequate housing or homelessness (35).^2^ In this context, the essential strategy that is assigned to the robot is to prevent self-neglect on the part of people suffering from dementia.

The tasks of reminding about and monitoring medication intake were mentioned as high level priority functionalities, which must be implemented into the robotic assistant. Douglas et al. emphasized that accidental injuries from errors in drug self-administration are more likely to occur for people with dementia than injury from fires/burns and wandering. These errors were attributed to cognitive deficits, sensory or physical problems with dispensers, or the complexity of the treatment regimen (31).

Interesting findings were recorded with regard to questions about reminding of meal or drink times and keeping in touch with family and friends. Elderly people with cognitive impairment reported that these functionalities are not necessary to be implemented. On the contrary, caregivers and medical staff argued that the robots must have the ability to perform these tasks. The differences may be a result of varied perceptions of disability. It is hard for early dementia patients to agree that they must be reminded about the simplest and basic human needs, which for older adults can be found as associated with the idea of “giving up” or of admitting defeat (36).

An important aspect of ensuring home security for persons with cognitive impairment is to prevent visits of unknown persons. It is especially important to avoid intrusions into the household. Older people, especially those with cognitive impairment, are an easy target for thieves. Hence, recognizing strangers is such an important functionality for caregivers and medical staff with regard to ensuring user safety.

Regarding the functionalities such as finding and reaching for things to bring them to patients, they are highly important for the primary users and of medium importance for the caregivers and medical staff. It is a very convenient feature, which makes life easier but otherwise may lead to the reduction of stimulating patients to remember. The same finding was observed in other studies where cognitively impaired patients were also interested in the functionalities offered by an assistive robot, like the object finding system (12).

These functionalities for monitoring and supervising the health and safety of the user are at a high level of priority and should be provided autonomously.

The other important ability of the robot is to provide cognitive stimulation to the potential user in two ways.

First, by engaging in physical and psychological activity and second, by providing cognitive entertainments. According to the medical personnel and caregivers’ opinions, this ability should be provided proactively but the potential users’ opinions were divided with a proportion 60/40 in the direction of on-demand. Recent research has been increasingly focused on the cognitive system, which is necessary to boost human cognitive capabilities or at least to inhibit the progress of cognitive decline (37–39).

Furthermore, traditional cognitive training with paper and pencil usually requires experienced instructors (40), but those qualified instructors may be unavailable for all in need in the coming years. For these reasons, opportunities for communication with others often decrease with advanced age and poor communication environment is linked to impaired cognitive function in the elderly (41). Thus, robotic assistance is a novel strategy to improve cognitive functions and prevent cognitive decline.

Communication is essential to maintain motivation (42).

Living with a human-type communication robot may not only improve cognitive functions but also provide beneficial outcomes for daily activities, morbidity, and mortality in the elderly (38).

Communication based on voice commands was chosen as a high priority and the simplest way of human–robot interaction. This type of communication is natural for man and it requires no additional effort in this field. Respondents indicate that robotic assistants must be able to talk in response to the user regarding its current task/state and should also continuously listen to the user for commands. Reciprocity is an important factor in human–human interaction, so it can be expected that it should also play a major role in human–robot interaction (43). It is very important that potential user and robot engage not only in natural interaction but also in alternative means of non-verbal communication such as simple gestures, remote control, and touch screen, which were chosen. Our findings are in line with previous studies (44). Additionally, Frennert et al. identified that for some users subtitles should be displayed on the screen while the robot is speaking. This solution would help to avoid misunderstandings when the robot is speaking and the robot’s voice is hardly audible.

We also found positive attitude toward socio-emotional interaction between the elderly and robots, which was expressed in a request that the robotic assistant should be able to understand the psychological state of the user and then provide positive affective impact. Socio-emotional interaction poses key requirements with which to create sustainable relationships between the elderly and robots, and this type of interaction will enhance the users’ acceptance and encourage the adoption of the assistive robotic system (45). The feeling of loneliness may negatively impact a person’s ability to act and engage in everyday activities. Robotic assistants could be a substitute for reducing loneliness of elderly people in two ways with direct and indirect impact. The direct influence means positive emotional human–robot interaction, or the robot as a friend. The indirect effect can be provided by stimulating the user to keep in touch with family and friends. Svanstrom et al. found that without the presence of others, a person with dementia seemed to lack initiative and experienced difficulties in managing everyday life. The authors highlight the importance of the presence of caregivers for those who live alone with dementia, who address the needs for socialization and do not solely focus on specific tasks or physical needs (46). In our study part of caregivers confessed that the burden of caring for their relatives and unmet needs themselves provoke frustration and unfriendly attitude toward their relative. They considered a robotic assistant to be a solution to improve relationships with their relative. This is a very important aspect, which must be considered because maintaining toxic relationship when the supporting person becomes frustrated may terminate a caregiving relationship (25).

Considered less important, the functionalities, which were assigned medium level priority were connected with performing basic daily activities, such as preparing food, dressing. They considered that robots might be helpful by reaching, grasping, finding things, and maintaining the sequence of tasks, which helps them compensate for their impairment. This result is not surprising, as demonstrated in many previous studies (44, 47, 48).

Surveys also identified robot capabilities with low importance for the Polish respondents. They are related to daily relaxation activities, like reminding about TV programs or these relating to assistance in shopping and cleaning the house. However, interesting findings were observed when comparing Polish and Spanish respondents. The functionality of cleaning the house was identified as high-value for Spanish respondents contrary to the Polish ones. Also, other functionalities listed above, which are not important for Polish users, are more important for Spanish ones. Here, the national and cultural differences are the most visible, which may stem from different lifestyles. Research performed on populations from well developed countries indicated that the perception of the robot as enjoyment or entertainment increases the inclination to use it (49, 50).

Another aspect of the requirements for the robot is its appearance. According to the literature, the robot’s appearance must be appropriately correlated with its functionality. It influences how people appraise the capabilities of the robot. It means that if a robot looks like a toy or pet, it will be treated as a source of entertainment and nobody will expect that such a robot is responsible for monitoring human health (51). On the other hand, humanoid robots are also undesirable (11, 52, 53). Summarizing, it is unacceptable and may finally lead to a market failure of a robot when its appearance is not consistent with its functionality. That is why it was important for us to find our target group expectations about robot capabilities or functionalities with regard to its appearance. The results of our surveys show that the majority of the respondents would like a robotic assistant to have an anthropomorphic appearance (woman or neutral) with positive emotionally expressive face. Most of the respondents think that the robotic assistant should be shorter than the user and the best height is approximately chest height. It confirms findings from other studies where most of the respondents would like the robot to have neutral/genderless (44) or more realistic sophisticated feminine appearance, which is more appropriate in expressing basic emotions (54).

The main issue in developing new robot is its acceptance. If cooperation of people with robots is to be successful, they need to be accepted by the target group. Even the best robot with many functionalities but without social acceptance is worthless. According to one of the definitions (55), acceptance may be described as the willingness to incorporate the robot into personal life. So, it is subjective users’ perceptions of what robots are, how they work, and what they can or cannot do that will determine how they are perceived and finally accepted (56). Many of individual factors such as age, gender, education, cognitive ability, culture, needs, experience with technology, and the level of anxiety affect the acceptability of robots (57).

In the general context of the acceptance of robots and of the prioritization of the user requirements in relation to robotic assistants, we did not find differences in terms of age and gender. In the literature, we can find that the willingness to use robots by elderly people depends on the context. When people were asked whether they could imagine living on a daily basis with robots, the most positive responses were from young adults and the least positive from adults over 65 years. On the other hand, when asked the question in a different way whether they would accept a robot to help them gain independence when they could no longer handle everyday tasks, acceptance increased particularly in the oldest age range (52, 57). In the same line of reasoning, we noted that over 80% of the respondents accept robotic assistants as a way to remain healthy and safe in their own homes. The high level of acceptance in our study also confirmed the findings that the acceptability of this specific solution is probably influenced by the coping strategies (58, 59), which is a matter of perceived self-efficacy. Pino et al. (49) and Bandura (60) demonstrated that participants with MCI and caregivers had more positive perception of the usefulness of robots than healthy older adults. Cesta et al. emphasized that it is important to address the issue of how frailty is perceived, with reference to both health in general and fear of cognitive weakening. More specifically, it can influence the evaluation of potential aid in everyday life, namely the robotic assistant. In the literature, it can be found that MCI persons did not consider themselves as potential users. They stated that a robot could be potentially useful either for themselves in the future or for other older adults suffering from frailty, loneliness, and disability (12, 36, 61).

The differences in the readiness to use a robotic assistant between elderly people are noticeable. Some people want to be independent and not be a burden to other people, and for them, a robot would be an ideal solution. This group of elderly people expresses readiness to accept a robotic assistant to help them (62). Others are now convinced that they do not need or want a robot. They considered that it is the duty of the family, the state to provide them with constant care, and not leaving them in the care of a robot. This attitude could be considered as passive resistance (12, 63). Many studies indicated possible factors underlying this attitude (12, 63–66).

On the whole, this attitude is associated with the type of personality and sometimes with a strong sense of fear of something new. In our study, above 80% of respondents accept a robot as a caregiver but being presently ready to remain in the care of a robot is expressed by only half of them. Among our respondents, it can be observed that postponed readiness probably stems from the lack of familiarity with technology, which can be explained by the fact that almost 70% cognitively impaired respondents and almost 84% of caregivers expressed the need to attend training sessions (minimum 3–4 training sessions) before they agree to leave the users alone with a robotic assistant. Lack of familiarity with technology can be a reason for people to feel uncertain about robots (67).

Direct experience of the usefulness of assistive devices can change older people’s attitudes from considering assistive devices unnecessary to considering them very useful (68). Promotional strategies that present a product as comfortable and facilitating the life of the consumer will cause people to want it and consequently feel that these are needed. A good example of such a strategy is presented by companies introducing new innovative products to the market. Referring to the robot, considering that people tend to have limited knowledge, it is good to show them the robot’s functionalities and how it can help them in daily activities, as it has been demonstrated by Broadbent et al. (57), who showed that, in individuals, unfamiliar with robotic healthcare, promoting appropriate expectations may be both feasible and effective.

Acceptability has multidimensional aspects and requires a broad approach but may be modified. An interesting finding was observed during one study where, at first, most of the participants in the focus groups were negatively disposed toward the idea of robots but after 1 h or so, after finding out more about the robots, some of them changed their mind and thought robots might be “good for others but not themselves” (44). Koay et al. found peoples’ preferences regarding robots changed over a period of time during a 5-week trial as participants grew familiar with robots (69).

The strength of our study is that the results were obtained from two autonomic centers and with the use of mixed methods with a wide range of stakeholders. Broadbent et al. emphasized that, to adjust the design of assistive robots, one has to assess the expectations and needs of a wide-range stakeholders. Most studies conducted in the dementia care context have only focused on one stakeholder group (21). Other included more group perspectives but involved a small sample size (49). A small sample size reduces both the chance of detecting true effects and the likelihood that a statistically significant result reflects a true effect (70). The minimum sample size for using maximum likelihood estimation is 100 (71) and this requirement was fulfilled in. our study (264 of the respondents). Additionally, the collected data came from different countries and provided an opportunity to identify different social cultural aspects in this field of study. This assisted researchers in identifying insights from different socio-cultural points of view (72).

It is worth mentioning that for the purpose of data, gathering focus groups were conducted. This type of methodology is considered an appropriate technique for preliminary data gathering; it makes it possible to obtain a wider range of experiences and ideas (73, 74). The selected groups held discussions based on their knowledge where appropriate. In the general context of dementia, caregivers and medical staff have the greatest knowledge to establish needs of individuals with cognitive impairment in a multidimensional aspect.

Two types of questions in surveys were used like closed-ended and open-ended. This methodology avoids bias because using only closed-ended question might force one to choose some response items even if respondents do not support exactly what the author thinks (12).

The main limitation of this study lies in the conceptual perception of an assistive robot rather than actual use in a sample of elderly people with cognitive impairment.

## Conclusion

It will be a long time before a robot can be capable of supporting multiple activities in a physical manner in the home of an elderly person in order to enhance their independent living capacity. The results from our study might contribute to a better understanding of the users’ needs and system requirements for the development of a robot intended to support older adults with cognitive impairment at home and their informal caregivers.

The development of robotic assistants in the general context of dementia requires both the understanding of the needs of the persons with cognitive impairment and the intensification of dissemination activities to shape a positive image of assistive robots.

In the literature, we can observe how the attitude toward technology has changed in the direction of adopting new solutions and perceiving them as something useful, pleasant. So, this phenomenon of positive change should gain pace in the direction of robotics seen as something natural and not stigmatizing people with infirmities.

Our results hold a promise that people with cognitive impairment are increasingly willing to take the robot to their home. Thus, assistive robots must meet the challenges as a caregiver of proven reliability, usability, and efficiency.

In view of the observations presented above, this study allows us to conclude that early dementia persons are increasingly willing to entrust themselves to the care of a robot that will meet the needs they have specified.

Nevertheless, longitudinal studies in the application of assistive robots are required.

## Ethics Statement

Local ethics committee of Medical University of Lublin. Local ethics committee of Fundacio ACE. Project ethics advisory board.

## Author Contributions

AK—substantial contributions to the conception or design of the work, analysis, or interpretation of data for the work, drafting the work, and final approval of the version to be published. SS—interpretation of data for the work, revising it critically for important intellectual content, and final approval of the version to be published. CR, NT, JH-F, JR, US, KG-A, DS-S—design of the work, revising it critically for important intellectual content, and final approval of the version to be published. KR—substantial contributions to the conception revising it critically for important intellectual content and final approval of the version to be published. All authors agreed to be accountable for all aspects of the work in ensuring that questions related to the accuracy or integrity of any part of the work are appropriately investigated and resolved.

## Conflict of Interest Statement

The authors declare that the research was conducted in the absence of any commercial or financial relationships that could be construed as a potential conflict of interest.

## Footnotes

^1^http://www.who.int/mediacentre/factsheets/fs362/en/.

^2^https://ncea.acl.gov/faq/.
